# Serum biomarkers and postherpetic neuralgia in herpes zoster patients with immunocompetent

**DOI:** 10.3389/fneur.2025.1623229

**Published:** 2025-08-04

**Authors:** Huili Liu, Yanrong Yuan, Jun Wang, Yan Zhang, Yongxing Yan

**Affiliations:** Hangzhou Third People’s Hospital, Hangzhou, China

**Keywords:** herpes zoster, neuralgia, immunocompromised, immunocompetent, serum, markers

## Abstract

**Background:**

Postherpetic neuralgia (PHN) typically occurs in immunocompromised patients. However, there is a lack of prospective studies involving large samples of immunocompetent individuals. A prospective study was conducted to ascertain the association between serum levels of various markers and the occurrence of PHN in acute herpes zoster (HZ) patients with immunocompetent.

**Methods:**

A total of 887 acute HZ patients with immunocompetent who were admitted to Hangzhou Third People’s Hospital between April 2021 and December 2023 were selected. Peripheral venous blood at their initial visit was collected and the levels of various serum markers were detected. According to whether PHN occurred or not during follow-up, the participants were divided into PHN group and non-PHN group. Multivariate logistic regression analysis was used to screen the influencing factors of PHN.

**Results:**

Two hundred and seventeen cases (24.5%) developed PHN. Compared with the non-PHN group, age, numerical rating scale (NRS) scores, levels of C reactive protein (CRP) and homocysteine (HCY) in PHN group were significantly increased (*p* < 0.01), while CD3^+^ (*p* < 0.05), CD4^+^ (*p* < 0.01), CD4^+^/CD8^+^ ratios (*p* < 0.05), albumin levels (*p* < 0.01), and albumin/globulin (A/G) ratios (*p* < 0.05) were significantly decreased. Multivariate logistic regression analysis showed that age, NRS scores, levels of CRP, and HCY were independent risk factors for PHN among acute HZ patients with immunocompetent. ROC curve analysis showed that the sensitivity and specificity of combined with age, NRS scores, CRP and HCY levels in predicting PHN were 68.4 and 74.1%, respectively.

**Conclusion:**

Age and the severity of pain at the time of onset were also the risk factors for PHN in acute HZ patients with immunocompetent. The levels of serum CRP and HCY as potential biomarkers may have certain reference value for predicting the occurrence of PHN in acute HZ patients with immunocompetent.

## Introduction

Herpes zoster (HZ) is an infectious skin disease caused by reactivation of the varicella zoster virus (VZV) latent in the host, which can cause skin blisters, erythema with pain, and other symptoms. Epidemiological studies have found that the incidence rate of HZ is gradually increasing. Patients with HZ often have various complications, of which postherpetic neuralgia (PHN) is the most common complication. The severity of pain varies from mild to severe, lasting for a long time, and seriously affecting the quality of daily life of patients.

In contrast to the high incidence of PHN, the cure rate of PHN is low. Previous studies showed that more than half of the PHN patients had no significant improvement in symptoms after treatment (1). However, administering regular antiviral drugs and active interventional treatment in the early stages of HZ can prevent the occurrence of PHN (2). Therefore, many scholars began to search for risk factors of PHN in recent years. However, most previous studies were of retrospective design, and predictive indicators are mostly limited to clinical factors, with few objective indicators (3–6). Objective assessment tools such as VZV skin test response and infrared thermography have been found to predict the occurrence of PHN in patients with HZ (7, 8). It is essential to find more easily obtainable objective indicators to predict the PHN occurrence for clinicians. Among them, serological indicators are the most important. Secondly, HZ patients with immunocompromise have a stronger willingness to seek medical attention due to their higher risk of developing PHN (5, 9, 10). However, there are still many immunocompetent individuals who experience HZ in clinical practice, and many of these patients often neglect timely treatment, leading to the development of PHN. However, there is limited research on the occurrence of PHN in these HZ patients. Therefore, this study aims to prospectively investigate whether the levels of serum biomarkers in acute HZ patients with immunocompetent can be used to predict PHN occurrence and guide treatment. This will provide clinicians with early intervention references.

## Materials and methods

### General information

A total of 887 acute HZ patients with immunocompetent who were admitted to Hangzhou Third People’s Hospital between April 2021 and December 2023 were selected. Baseline clinical characteristics such as gender, age, herpes location, pain score, comorbidities, and the intervals between onset and treatment were recorded. This study was approved by the Medical Ethics Committee of Hangzhou Third People’s Hospital (No. 2021KA013).

*Inclusion criteria*: (1) Age ≥18 years; (2) patients with typical clinical manifestations of HZ, meeting diagnostic criteria for HZ (11) and PHN (12); (3) patients without receiving formal treatment; (4) onset within 14 days; and (5) able to attend hospital follow-up or receive telephone follow-up after discharge.

*Exclusion criteria*: (1) Patients with immunocompromised status, including impaired immune function, mainly including solid organ malignant tumors, hematological malignancies, solid organ transplantation, hematopoietic stem cell transplantation, HIV infection, congenital immunodeficiency, long-term use of immunosuppressants, etc. (13); (2) patients with dementia, aphasia, or language disorders; (3) patients with zoster sine herpete; (4) pregnant or lactating women; (5) patients declining follow-up participation.

### Detection of serum markers

All enrolled patients underwent fasting venous blood sampling on the morning of the second day after admission for testing blood routine [white blood cell (WBC), red blood cell (RBC), hemoglobin (Hb), platelet (PLT), neutrophils, lymphocytes, etc.], C-reactive protein (CRP), blood lipids [triglyceride (TG), total cholesterol (TC), low-density lipoprotein cholesterol (LDL-C), high-density lipoprotein cholesterol (HDL-C)], liver function (albumin, globulin, total protein, bilirubin, total bile acids, etc.), kidney function [blood urea nitrogen (BUN), creatinine (Cr)], blood glucose, uric acid, homocysteine (HCY), thyroid function (T3, T4, TSH, etc.), T lymphocyte subpopulation levels (CD3^+^, CD4^+^, CD8^+^, CD19^+^, CD56^+^), immune function (C3, C4, IgA, IgG, IgM), etc. These tests were performed using a 7,600–020 fully automatic biochemical analyzer (Hitachi, Japan), a BD FACSCalibur flow cytometer (BD, United States), strictly following the operating procedures of the machines and the requirements of the reagent kits for the determination of blood markers.

### Pain assessment

The numerical rating scale (NRS) is used to assess the pain intensity through patient self-rating on a 10-point scale, where 0 represents no pain and 10 represents the most severe pain. 1–3: mild pain (tolerable without functional impairment); 4–6: moderate pain (affects sleep and requires analgesic intervention); 7–10: severe pain (unbearable with significant functional limitation). All patients underwent NRS scores by a dedicated physician both on the day of admission and during follow-up.

### Follow-up

All enrolled patients were followed up by phone or through outpatient visits to record their pain after herpes healing, and the diagnosis of PHN was determined based on diagnostic criteria (12). All patients were divided into PHN group and non-PHN group based on PHN occurrence status.

### Statistical analysis

Statistical analysis was performed using SPSS 27.0 software. Normality of measurement data was assessed by the Kolmogorov–Smirnov test, with normally distributed data expressed as mean ± standard deviation (‌x̄ ± SD). Independent sample *t*-test was used for single-factor analysis of normally distributed data, while non-normally distributed data were presented as median (interquartile range) [M (Q1–Q3)] and analyzed using the Mann–Whitney *U* test. Categorical data was expressed as counts (percentage) [*n* (%)], with between-group comparisons performed by *χ*^2^ test or Fisher’s exact test. Firstly, univariate analysis is applied to identify the potential risk factors of PHN. Subsequently, multiple logistic regression analysis was applied to determine independent risk factors. Predictive performance of each indicator was evaluated using receiver operating characteristic (ROC) curves and area under the curve (AUC), the Youden index to determine the cut off value. A two-sided *p*-value <0.05 was considered statistically significant.

## Results

### Baseline clinical characteristics of patients in PHN and non-PHN groups

All 887 enrolled patients with acute HZ completed follow-up. During follow-up period, 670 patients, including 311 males (46.4%) and 359 females (53.6%), showed no neuralgia (non-PHN group), with an average age of 58.5 ± 14.8 years. The interval between onset and treatment was 5.3 ± 2.6 days; NRS scores are 2.4 ± 1.5 points. There were 217 patients (217/887, 24.5%) in PHN group, including 111 males (51.2%) and 106 females (48.8%), with an average age of 63.8 ± 16.9 years. The interval between onset and treatment is 5.3 ± 2.7 days; the NRS scores are 3.7 ± 1.9 points. Compared with the non-PHN group, patients in PHN group were older and had higher NRS scores (*p* < 0.01). However, there was no significant difference in gender and the interval between onset and treatment between the two groups (*p* > 0.05, Table 1).

**Table 1:** Comparison of baseline clinical characteristics between PHN and non-PHN groups.

Characteristics	Non-PHN group (*n* = 670)	PHN group (*n* = 217)	*t*/*χ*^2^	*p*
Age (X ± SD) (years)	58.5 ± 14.8	63.8 ± 16.9	4.410	0.000
Gender [*n* (%)]				
Male	311 (46.6)	111 (51.2)	1.4	0.2
Female	359 (53.6)	106 (48.8)	73	25
Comorbidities [*n* (%)]				
Hypertension	199 (29.7)	77 (35.5)	2.557	0.110
Type 2 diabetes	64 (9.6)	21 (9.7)	0.003	0.957
Coronary heart disease	16 (2.4)	5 (2.3)	0.005	0.944
Atrial fibrillation	3 (0.4)	2 (0.9)	0.657	0.418
Stroke	14 (2.1)	4 (1.8)	0.050	0.823
Herpes site [*n* (%)]				
Head and neck	344 (51.3)	116 (53.5)	0.293	0.588
Chest and back	106 (15.8)	35 (16.1)	0.012	0.914
Waist and abdomen	119 (17.8)	34 (15.7)	0.503	0.478
Limb	81 (12.1)	26 (12.0)	0.002	0.966
Disseminated	20 (3.0)	6 (2.8)	0.028	0.867
Interval between onset and treatment (X ± SD) (days)	5.3 ± 2.6	5.3 ± 2.7	0.080	0.936
NRS scores	2.4 ± 1.5	3.7 ± 1.9	10.38	0.000

The main comorbidities in both non-PHN and PHN groups were hypertension (29.7% vs. 35.5%), type 2 diabetes (9.6% vs. 9.7%), and coronary heart disease (2.4% vs. 2.3%). No statistically significant differences existed in comorbidity profiles between groups (*p* > 0.05).

In 670 non-PHN patients, herpes zoster distribution was predominately head and neck (344 cases, 51.3%), followed by waist and abdomen (119 cases, 17.8%), chest and back (106 cases, 15.8%), limbs (81 cases, 12.1%), and disseminated herpes (20 cases, 3.0%). In 217 PHN patients, the distribution was: head/neck (116 cases, 53.5%), chest and back (35 cases, 16.1%), waist and abdomen (34 cases, 15.7%), limbs (26 cases, 12.0%), and disseminated (6 cases, 2.8%). there were 116 cases, 35 cases, 34 cases, 26 cases, and 6 cases of herpes distributed in the head and neck, back, waist and abdomen, limbs, and disseminated, respectively. No significant difference in the location of herpes was observed between groups (*p* > 0.05, Table 1).

### Comparison of serum markers levels between two groups

Univariate analysis revealed that compared with the non-PHN group, patients in the PHN group exhibited significantly higher levels of CRP (*p* < 0.01) and HCY (*p* < 0.01). Conversely, significant reductions were observed in: CD3^+^ (*p* < 0.05), CD4^+^ (*p* < 0.01), CD4^+^/CD8^+^ ratios (*p* < 0.05), albumin levels (*p* < 0.01), and A/G ratios (*p* < 0.05). No significant differences in liver function, kidney function, blood lipids, thyroid function, C3, C4, IgA, IgG, IgM levels were observed between groups (all *p* > 0.05, Tables 2, 3).

**Table 2:** Comparison of the levels of serum markers related immune function between two groups (X ± SD).

Characteristics	Non-PHN group (*n* = 670)	PHN group (*n* = 217)	*t*	*p*
Cellular immune level				
CD3^+^ (×10^9^/L)	931.0 ± 436.9	832.1 ± 401.8	2.596	0.010
CD19^+^ (×10^9^/L)	179.1 ± 120.1	176.2 ± 145.0	0.252	0.801
CD8^+^ (×10^9^/L)	347.1 ± 211.4	319.9 ± 186.3	1.498	0.135
CD4^+^ (×10^9^/L)	533.1 ± 263.2	460.7 ± 248.6	3.140	0.002
CD56^+^ (×10^9^/L)	264.8 ± 232.5	286.3 ± 209.2	1.064	0.288
CD4^+^/CD8^+^ ratios	1.9 ± 1.3	1.6 ± 0.8	2.362	0.019
Humoral immune level				
C3 (g/L)	1.2 ± 0.4	1.2 ± 0.2	0.403	0.687
C4 (g/L)	0.4 ± 0.1	0.4 ± 0.2	1.834	0.067
IgA (g/L)	2.3 ± 1.6	2.5 ± 1.7	0.997	0.319
IgG (g/L)	12.0 ± 4.5	11.8 ± 2.9	0.539	0.590
IgM (g/L)	1.0 ± 0.7	1.1 ± 0.8	0.713	0.476

**Table 3:** Comparison of the levels of serum markers between two groups (X ± SD).

Characteristics	Non-PHN group (*n* = 670)	PHN group (*n* = 217)	*t/Z*	*p*
WBC (×10^9^/L)	6.1 ± 3.4	5.8 ± 2.1	1.119	0.263
RBC (×10^12^/L)	4.4 ± 0.6	4.3 ± 0.5	0.233	0.816
Hb (g/L)	133.4 ± 17.7	133.2 ± 15.4	0.110	0.913
PLT (×10^9^/L)	199.6 ± 56.1	191.9 ± 54.6	1.781	0.075
Lymphocytes (×10^9^/L)	1.4 ± 0.7	1.5 ± 1.3	0.743	0.458
Neutrophils (×10^9^/L)	4.0 ± 1.9	3.8 ± 1.7	1.196	0.232
CRP (mg/L)	2.8 (0.75,7.15)	4.5 (2.0,10.50)	4.413	0.000
Albumin (g/L)	37.5 ± 4.3	36.5 ± 3.1	3.084	0.002
Globulin (g/L)	27.1 ± 3.6	27.7 ± 3.8	1.789	0.074
A/G ratios	1.4 ± 0.4	1.3 ± 0.2	2.294	0.022
Total protein (g/L)	64.6 ± 6.0	63.9 ± 6.5	1.421	0.156
Glutamic-pyruvic transaminase (U/L)	22.3 ± 19.5	21.4 ± 17.3	0.595	0.552
Glutamic oxaloacetic transaminase (U/L)	20.5 ± 10.4	20.5 ± 8.7	0.022	0.982
Creatine kinase (U/L)	75.9 ± 48.0	76.3 ± 49.3	0.130	0.896
Total bilirubin (μmol/L)	12.0 ± 5.9	11.3 ± 4.8	1.713	0.087
Direct bilirubin (μmol/L)	4.1 ± 2.2	4.0 ± 3.1	0.452	0.651
Indirect bilirubin (μmol/L)	8.0 ± 5.1	7.9 ± 7.1	0.320	0.749
β2-microglobulin (mg/L)	2.0 ± 0.8	2.2 ± 1.2	1.758	0.079
BUN (mmol/L)	5.4 ± 4.3	5.6 ± 4.6	0.199	0.843
Cr (μmol/L)	67.8 ± 35.3	66.9 ± 31.2	0.321	0.748
Glucose (mmol/L)	6.5 ± 4.8	6.3 ± 2.0	0.593	0.553
Uric acid (μmol/L)	283.3 ± 120.3	277.4 ± 90.1	0.656	0.512
HCY (μmol/L)	11.2 ± 7.1	14.1 ± 9.1	4.472	0.000
T-ch (mmol/L)	4.3 ± 0.9	4.4 ± 0.9	1.062	0.289
LDL-C (mmol/L)	2.7 ± 0.8	2.8 ± 0.8	0.930	0.353
HDL-C (mmol/L)	1.3 ± 0.4	1.3 ± 0.3	0.891	0.373
TG (mmol/L)	1.2 ± 0.8	1.2 ± 0.9	0.430	0.668
TT3 (nmol/L)	4.0 ± 1.3	4.0 ± 1.1	0.010	0.993
TT4 (nmol/L)	17.0 ± 7.3	18.0 ± 16.0	1.175	0.240
TSH (mIU/L)	1.5 ± 1.3	1.5 ± 1.4	0.214	0.831
K^+^ (mmol/L)	4.0 ± 0.4	4.2 ± 2.8	1.645	0.100
Na^+^ (mmol/L)	139.9 ± 8.6	140.0 ± 5.9	0.067	0.947
Cl^−^ (mmol/L)	102.6 ± 3.4	102.2 ± 3.6	1.561	0.119
Ca^++^ (mmol/L)	2.3 ± 0.5	2.3 ± 0.6	0.995	0.320
Fe^+++^ (μmol/L)	12.7 ± 6.0	13.3 ± 9.5	0.864	0.388

### Analysis of independent risk factors for PHN in acute HZ patients with immunocompetent

Multivariate logistic regression analysis was conducted with the presence or absence of concurrent PHN as the dependent variable and *p* < 0.05 factors in univariate comparisons (age, NRS scores, CRP, HCY, CD3^+^, CD4^+^, CD4^+^/CD8^+^ ratios, albumin level, A/G ratios) as independent variables. The results showed that age, NRS scores, CRP and HCY levels were independent risk factors for PHN in acute HZ patients with immunocompetent. In contrast, CD3^+^, CD4^+^, CD4^+^/CD8^+^ ratios, albumin levels, A/G ratios showed no significant association (all *p* > 0.05). Multivariate logistic regression analysis indicated that after adjustment for confounding factors such as age, gender, NRS scores, and lesion location, CRP and HCY retained statistical significance as independent risk factors for PHN in acute HZ patients with immunocompetent (Table 4).

**Table 4:** Multivariate logistic regression analysis of risk factors for PHN in acute HZ patients with immunocompetent.

Variable	*β*	*Wald*	*p*	*Exp.*	95%CI
Age	0.018	5.509	0.019	1.018	1.003–1.034
NRS scores	0.480	49.042	0.000	1.616	1.413–1.849
CRP	0.021	4.123	0.042	1.021	1.001–1.042
HCY	0.036	6.329	0.012	1.037	1.008–1.067
Albumin	−0.020	0.300	0.584	0.880	0.749–1.010
A/G ratios	−0.985	2.299	0.129	0.373	0.104–1.334
CD3^+^	−0.002	2.515	0.113	0.998	0.996–1.000
CD4^+^	0.002	0.802	0.370	1.002	0.998–1.005
CD4^+^/CD8^+^ ratios	−0.383	3.659	0.056	0.682	0.461–1.009

**Figure 1:**
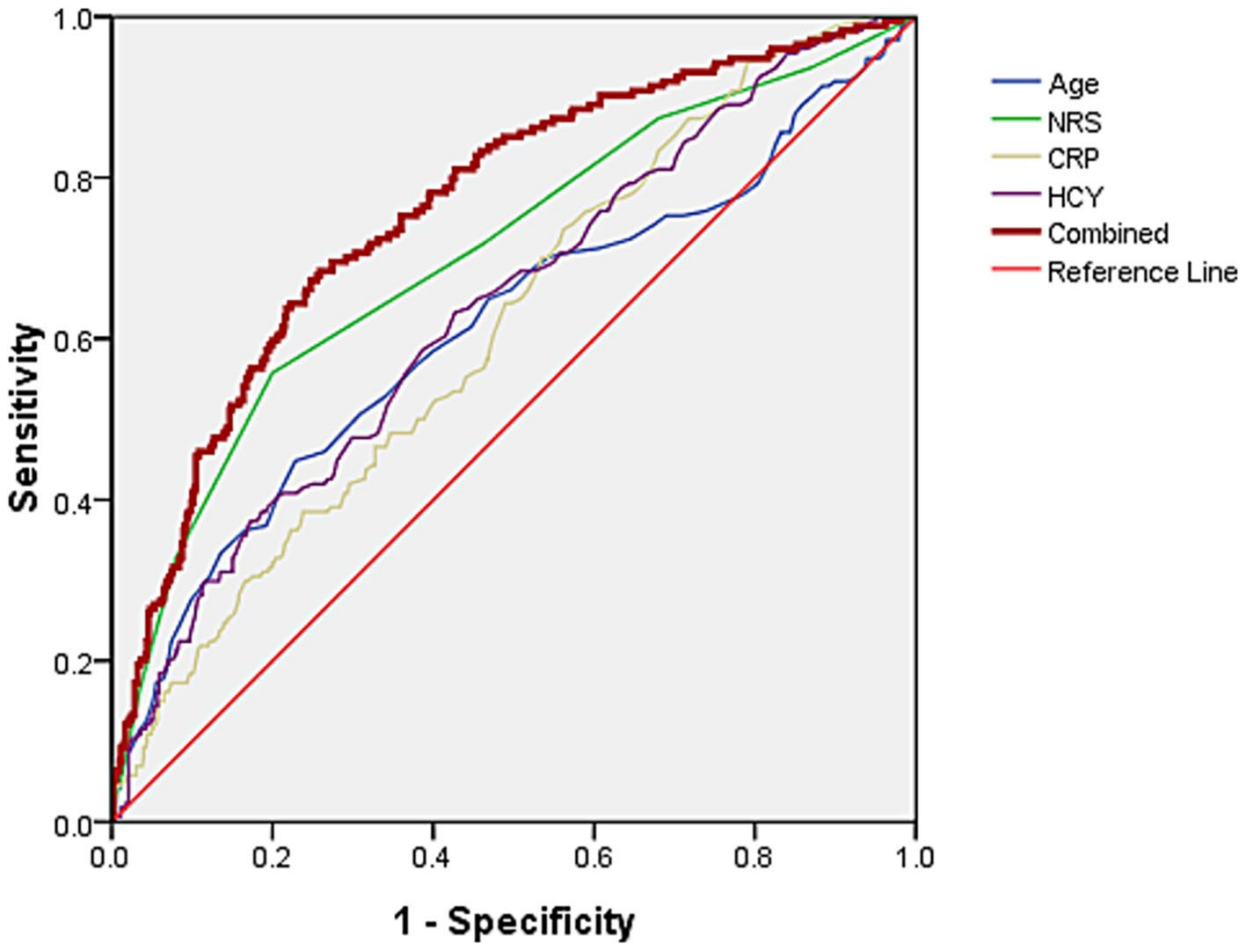
The AUC of age, NRS scores, and CRP and HCY levels predict concurrent PHN for acute HZ patients with immunocompetent were 0.612, 0.705, 0.615, and 0.637, respectively. The AUC of predicting PHN occurrence using combined these four indicators was 0.762, with sensitivity and specificity of 68.4 and 74.1%, respectively.

The area under the curve (AUC) values of age, NRS scores, CRP, and HCY levels for predicting subsequent PHN in acute HZ patients with immunocompetent were 0.612, 0.705, 0.615, and 0.637, respectively. A base on logistic regression predicted probabilities combined model incorporating these four predictors (when the cut off value of Age, NRS scores, CRP and HCY levels were 72.5 years, 4, 7.45 mg/L and 13.85 μmol/L, respectively.) demonstrated significantly enhanced discriminatory power (AUC = 0.762), with sensitivity and specificity of 68.4 and 74.1%, respectively (Figure 1).

## Discussion

Herpes zoster results from reactivation of VZV latent in sensory ganglia, and pain often occurs in the herpes distribution area. Literature indicates that 9–34% of HZ patients develop refractory PHN (14, 15). PHN incidence in immunocompromised individuals is high. Therefore, both clinicians and patients attach great importance to PHN occurrence in immunocompromised populations and will intervene it early. For immunocompetent individuals, the compliance is relatively low and treatment may be delayed. However, in the real world, the probability of developing neurological complications in individuals with immunocompetent is not low. For example, studies have found that immunocompetent patients with disseminated HZ had a higher incidence of PHN than immunocompromised cases (16). There are also reported cases of concurrent stroke and meningitis (17, 18). VZV is considered the increasingly common culprit of encephalitis in young and immunocompetent patients (19). Therefore, the complications should not be ignored in acute HZ patients with immunocompetent. The incidence rate of PHN in this study is 24.5% (217/887), which is similar to the proportion reported in the past (15, 20, 21), it may be related to the fact that all the patients included were hospitalized, and the diagnostic standard of PHN adopted was the Chinese diagnostic criteria. At present, many scholars believe that PHN is pain persisting for ≥3 months after the onset of herpes (11), but the diagnostic standard of PHN in China is defined as pain that lasting for 1 month or more after the healing of herpes (12). Due to different standards, the incidence rate of PHN is varied. If the definition of PHN used pain lasting ≥1 month after rash healing, it can help clinicians to intervene early, avoid the continuous development of pain, and help patients better to manage their pain, seek medical help earlier, improve treatment effectiveness.

The advantage of this study is that we recruited a relatively large number of patients, targeting immunocompetent populations, and analyzed more clinical and laboratory data. Based on the results of this study, clinicians should pay attention to the risk of developing PHN in elderly patients with acute HZ who experience significant pain on admission and have elevated blood CRP and HCY levels, they should actively intervene in advance. Age is a high-risk factor for the occurrence of PHN. This indicates that as age increases, elderly patients experience immune aging, with reduced cellular and humoral immune functions, leading to the spread of viral infections and severe nerve damage. At the same time, the ability of elderly individuals to repair neurological damage is weaker than that of young people, making them more prone to developing PHN (22–24). This is also consistent with the fact that HZ is a cellular immune related disease, any factor that leads to suppression of cellular immune function will increase the occurrence risk of HZ and PHN. There are studies showing a certain correlation between the clinical characteristics of HZ in acute phase and the onset of PHN. One meta-analysis found that odds ratio (OR) values for the correlation between prodromal and acute phase pain levels in patients with HZ and the risk of PHN were 2.29 and 2.23, respectively (25). This is consistent with the view that severe infections can cause more severe nerve damage, leading to PHN. Similar to immunocompromised individuals, we found that older age and higher NRS scores are independent risk factors for PHN in acute HZ patients with immunocompetent, further confirming that some clinical features of HZ patients in acute phase increase the risk of PHN. Hence, clinicians need to strengthen intervention for HZ patients with elderly, significant acute pain, even if they do not have immunocompromised.

The progression from acute HZ to PHN involves complex interactions between the virus, immune system and nervous system. Dysregulation of inflammatory response can lead to PHN. CRP levels can regulate the development of inflammation, promote chronic inflammation, and significantly increase in infectious diseases (26, 27). Previous studies have found that severe HZ patients have the highest levels of CRP, which may be due to the elevation of IL-6, which the main inducer of CRP production, leading to an overactive immune response (28, 29). Consistent with the results of our study, some studies also have shown a positive association between CRP levels and the pain in PHN patients (30, 31). HCY is a sulfur-containing amino acid in the human body, which is an essential intermediate product for the metabolism of methionine and cysteine. HCY has both pro-inflammatory and anti-inflammatory properties. The elevation of HCY levels may promote inflammation by increasing oxidative stress and inhibiting glutathione peroxidase, leading to cellular damage (32, 33). A study found a significant positive correlation between HCY levels in HZ patients with severe rash and those with chronic PHN. The increased HCY in HZ may reflect deficiencies in vitamin B6, B12, and folate, as well as an increase in inflammatory markers and oxidative stress. The pathogenic mechanism may be related to its neurotoxicity (30, 34). Our study found that HZ patients which concurrent PHN had significantly increased HCY levels, which is an independent risk factor for PHN, indicating that HCY and VZV may jointly participate in the neurological damage of HZ patients. Stein et al. (34) reported a statistically significant difference in HCY levels between HZ patients with and without PHN, HCY and CRP levels can be used as oxidative stress indicators to evaluate the antioxidant status of HZ. In addition, in a study of 43 HZ cases and 47 controls, it was also confirmed that elevated HCY and CRP levels were associated with increased inflammation in HZ patients (35). Nagasako et al. (36) classified the severity of HZ based on the number of papules, vesicles, or scabs, and confirmed that severe rash is a risk factor for long-term pain, in the severe rash group and PHN cases, uric acid and vitamin D levels were lower, while HCY and CRP levels were higher. Interestingly, HCY is associated with an increase in serum CRP concentration. CRP is an inflammation marker and is directly proportional to the severity of the disease, which may further reflect the same characteristics of inflammatory response in HZ (35). As Saguil et al. found that the long-term effects of inflammation and oxidative stress in the dorsal root ganglia can lead to neuronal damage and cell death, resulting in inflammatory hyperalgesia and neuropathic pain (37). This finding highlights the importance of timely screening for levels of CRP and HCY in acute HZ patients with immunocompetent. Early initiation of anti-inflammatory therapy may significantly influence the prognosis in these patients. In our study, univariate analysis revealed that patients in the PHN group exhibited significant reductions in CD3^+^, CD4^+^ and CD4^+^/CD8^+^ ratios, but the significance was lost in multivariate logistic regression analysis. They may be used as a confounding factor in multivariate analysis to affect the statistical results. This indicates that there are different risk factors for PHN in acute HZ patients with immunocompetent or immunocompromised.

This study has several limitations. First, as a single-center investigation exclusively enrolling hospitalized patients, selection bias may exist since individuals with milder acute HZ symptoms typically managed in outpatient settings were excluded. It may affect generalizability to outpatient or milder HZ cases. This likely contributed to the observed higher PHN incidence (24.5%) compared to community-based cohorts. Second, the main aim of this study is to evaluate the levels of serum markers in predicting PHN. Some important influencing factors, such as skin lesion severity, psychological status (e.g., anxiety, depression) and details of antiviral or analgesic treatment, being reported to be risk factors for PHN were not collected in the present study, Third, the serum markers levels of patients were not dynamically monitored in this study which may limits the understanding of their roles in the progression or resolution of symptoms.

In summary, PHN was prevalent among acute HZ patients with immunocompetent. Age, NRS scores, serum CRP and HCY levels are independent risk factors for PHN, and the combination of these four indicators has certain clinical reference value for predicting the occurrence of PHN. Clinicians should comprehensively evaluate the risk of concurrent PHN in elderly acute HZ patients who experience significant pain and have elevated levels of CRP and HCY. They should strengthen patient’s education, enable them to have a correct understanding of PHN and treat it positively, and provide timely standardized treatment to prevent the occurrence of PHN, improve patient’s long-term quality of life, reduce social burden. In the future, larger sample sizes and multicenter randomized clinical studies will be needed to evaluate the levels of serum biomarker changes in assessing the occurrence of PHN.

## Data Availability

The raw data supporting the conclusions of this article will be made available by the authors, without undue reservation.
